# Effects and Mechanism of Enhanced UV-B Radiation on the Flag Leaf Angle of Rice

**DOI:** 10.3390/ijms232112776

**Published:** 2022-10-24

**Authors:** Chengting Ling, Xiupin Wang, Zuran Li, Yongmei He, Yuan Li

**Affiliations:** 1 College of Resources and Environment, Yunnan Agricultural University, Kunming 650201, China; 2 College of Horticulture and Landscape, Yunnan Agricultural University, Kunming 650201, China

**Keywords:** UV-B, rice, flag leaf angle, anatomical structure, plant hormones, genes

## Abstract

Leaf angle is an influential agricultural trait that influences rice (*Oryza sativa* L.) plant type and yield, which results from the leaf bending from the vertical axis to the abaxial axis. UV-B radiation affects plant morphology, but the effects of varying UV-B intensities on rice flag leaves and the underlying molecular, cellular, and physiological mechanisms remain unknown. This experiment aims to examine the effect of natural light and field-enhanced UV-B radiation (2.5, 5.0, 7.5 kJ·m^−2^) on the leaf angle of the traditional rice variety Baijiaolaojing on Yuanyang terraces. In comparison with natural light, the content of brassinolide and gibberellin in rice flag leaves increased by 29.94% and 60.1%, respectively. The auxin content decreased by 17.3%. Compared with the natural light treatment, the cellulose content in the pulvini was reduced by 13.8% and hemicellulose content by 25.7% under 7.5 kJ·m^−2^ radiation intensity. The thick-walled cell area and vascular bundle area of the leaf pulvini decreased with increasing radiation intensity, and the growth of mechanical tissue in the rice leaf pulvini was inhibited. The flag leaf angle of rice was greatest at 7.5 kJ·m^−2^ radiation intensity, with an increase of 50.2%. There are two pathways by which the angle of rice flag leaves is controlled under high-intensity UV-B radiation. The leaf angle regulation genes *OsBUL1*, *OsGSR1*, and *OsARF19* control hormone levels, whereas the *ILA1* gene controls fiber levels. Therefore, as cellulose, hemicellulose, sclerenchyma, and vascular bundles weaken the mechanical support of the pulvini, the angle of the flag leaf increases.

## 1. Introduction

Ultraviolet-B (280–315 nm) is an essential component of sunlight. Even though it comprises only 0.5% of the total light energy reaching the Earth’s surface, it is of the most significance, due to its substantial impact on sessile plants [1,2]. Several morphological changes are caused by UV-B radiation in plants, including leaf thickening, petiole shortening, leaf area reduction, leaf curling, internode shortening, stomatal opening, increased axillary branching, reduced inflorescence elongation, and changes in root-to-shoot ratios [3,4,5,6,7]. Generally, these responses can be categorized into two types: photographic and stress reactions. The different types of responses are primarily determined by the intensity and duration of exposure [8]. The photomorphological development of plants is promoted by non-damaging ultraviolet-B (UV-B) light, and stress adaptation is largely mediated by the UV photoreceptor UVR8 [9]. It was demonstrated that *Arabidopsis* displayed a decrease in rosette diameter, a reduction in inflorescence height, and an increase in flower stems after long-term exposure to low-intensity UV-B radiation, indicating that chronic UV-B treatment resulted in a redistribution of the plant architecture [4]. Due to UVR8 pathway regulation, low-intensity UV-B radiation inhibits hypocotyl growth. Plant hypocotyl growth can be inhibited by UV-B radiation by inducing photomorphogenesis via UVR8 photoreceptors and DNA damage response. In *Arabidopsis thaliana*, mutants of endonucleases involved in Nucleotide Excision Repair (NER) were more likely to exhibit increased hypocotyl growth inhibition by UV-B in the DNA repair pathway [10,11]. Due to the increased level of DNA damage caused by UV-B in plants, the morphology of plants was also negatively affected.

Rice (*Oryza sativa* L.) is one of the world’s most widely grown crops, and its morphology determines its yield. In an analysis of five years of UV-B radiation treatment in Japanese mid-latitude rice, it was found that enhanced UV-B radiation inhibited the growth and development of rice grains [12]. In an experiment using rice supplemented with enhanced UV-B radiation in the southern United States, it was found that increased UV-B radiation decreased the panicle length, the number of branches per panicle, and the grain width, whereas spikelet sterility increased [13]. According to our previous experimental results in Yuanyang terraced fields in Yunnan Province, increased UV-B radiation affects grain structure, spike length, leaf morphology, and stem wall development in rice [14,15,16,17]. Despite this, little is known about how UV-B radiation affects the leaf angle, an imperative agronomic trait for cultivating ideal rice plants [18]. The leaf angle is determined by the curvature of the sleeper. As a result of the leaf’s upright position, the small angle between leaves, and the fact that the leaf receives light from both sides, the light capture rate is increased. In addition, the ventilation and light transmission conditions of the middle and lower leaves are enhanced, the photosynthetic capacity of the middle and lower leaves is enhanced, and organic matter accumulation is promoted [19,20]. Several studies have demonstrated that rice mutants deficient in *OsGLP1* (*Oryza sativa* germin-like protein1) showed an increase in leaf angle under solar radiation (including UV-B) and that germin-like proteins (GLPs) contribute to the adaptation of rice to UV-B exposure [21,22].

The formation of the leaf angle is due to the imbalance of cell division and growth on the adaxial and abaxial surfaces of the lamina [23,24,25,26]. In contrast, the development of the lamina is mainly influenced by BR (brassinolide), GA (gibberellins), and IAA (auxin) [19], which are significant contributors to leaf angle formation. GA and IAA often regulate leaf angle through their interaction with BR [20]. Several mutant plants have altered leaf angles due to the overexpression or genetic deletion of genes involved in hormone synthesis or signal transduction. The genetic analysis of these mutants has identified many genes, including *D2(dwarf-2)*, *OsBUL1*, *OsGSR1(GA-stimulated transcript gene)*, and *OsARF19*s(auxin response factor) [24,27]. Additionally, the rice lamina provides mechanical support for the flag leaf through its automatic organization. Changes mainly influence *ILA1(increased leaf angle1)* and *OsARF19* genes in the vascular bundle and sclerenchyma of the lamina [20,24]. It has been suggested, however, that increased UV-B radiation may be responsible for controlling the morphological responses of plants through the synthesis of hormones or the expression of genes associated with signal transduction. To better understand how rice flag leaf angles respond to UV-B radiation and how hormone synthesis and signal transduction work, it is necessary to investigate morphological responses to UV-B radiation.

A key enzyme involved in the biosynthesis of BR is encoded by the *D2* gene, which encodes CYP90D2. BR treatment significantly restored the phenotype of the dwarf mutant *ebisu dwarf* (d2), whose leaf angle was reduced considerably [28]. *Oryza sativa BRASSINOSTEROID UPREGULATED 1-LIKE 1* (*OsBUL1*) participates in BR signal transduction and positively regulates leaf angle [29]. By binding excess IAA to various amino acids, the *OsARF19* gene controls rice leaf angle [30,31]. *OsARF19* also modulates BR signaling. In *OsARF19-O1*-overexpressing plants, the BR biosynthesis genes *OsD2* (*CYP90D2*) and *OsDWARF* were significantly upregulated [28,32]. In the GA signaling pathway, *OsGSR1* plays a positive role. The silencing of this gene induces phenotypes such as leaf erection, increased levels of gibberellin synthesis gene *OsGA20ox2*, and an increase in endogenous gibberellin levels. By interacting with DIM/DWF1 (BRD2) enzymes in the BR biosynthetic pathway, *OsGSR1* also activates BR biosynthesis [33]. A putative mitogen-activated protein kinase kinase kinase (MAPKKK) is encoded by *ILA1*. *ILA1* mutant leaves have smaller vascular bundles, and cellulose, the main component of the cell wall, decreases, which reduces the pulvinus’ mechanical support, resulting in a larger leaf angle [34]. A significant increase in leaf angle was also recorded in plants overexpressing *OsARF19*, as well as a decrease in cellulose, which is the main component of the lamina sclerenchyma cell wall [31]. Despite extensive research on the role of hormones in regulating leaf angle, little is known about the role of ultraviolet-B radiation. By simulating the enhancement of UV-B radiation in the field, high-intensity UV-B radiation increased the included angle of rice flag leaves. Moreover, high-intensity UV-B radiation activated genes that affected leaf angle and inhibited the expression of the *ILA1* gene after high-intensity UV-B radiation. In response to enhanced UV-B radiation, brassinolide and gibberellin positively regulated the flag leaf angle, and auxin negatively regulated the flag leaf angle. This study supports the hypothesis that UV-B radiation regulates the angle and direction of flag leaves in rice by regulating cell division and elongation, mechanical organization, and cell wall biosynthesis in the pulvini.

## 2. Results

### 2.1. The Leaf Angle Was Increased by High-Intensity UV-B Radiation

**Figure 1:**
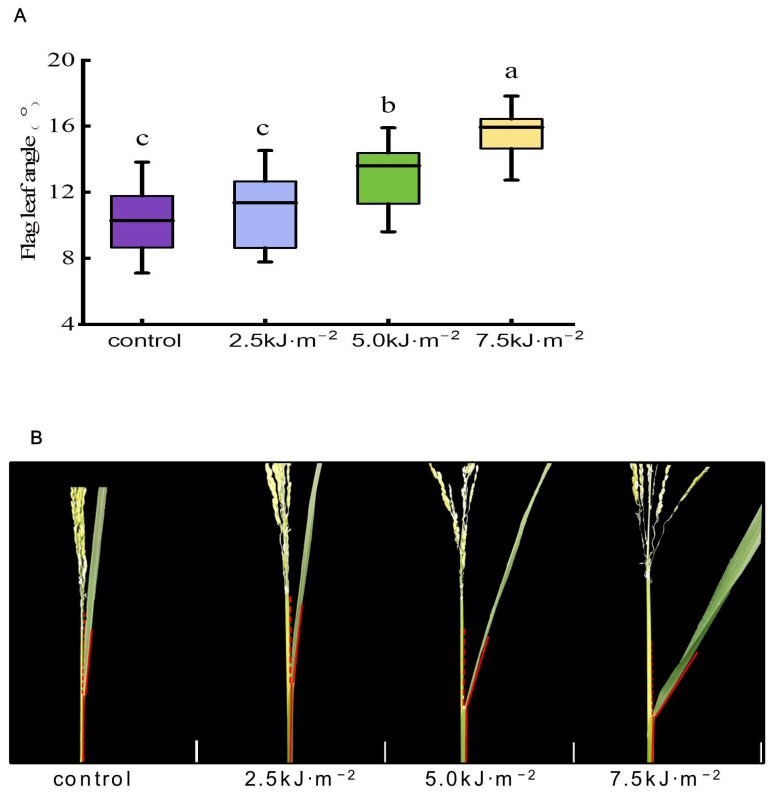
Effect of UV-B radiation on the angle of flag leaves in rice. (**A**) The leaf angle of rice flag leaves at the heading stage under natural light (control) and 2.5 kJ·m^−2^, 5.0 kJ·m^−2^, and 7.5 kJ·m^−2^ UV-B radiation intensity. The horizontal bar within the box represents the median. The top and bottom of the box represent 0.75 and 0.25 percentiles, respectively. The upper and lower whiskers extend to 1.5 times the interquartile range, with outliers shown as black dots. Error bars are SE (*n* = 20). Different letters indicate significant differences, *p* < 0.05, by one-way ANOVA. (**B**) The leaf angle morphology of rice flag leaves at the heading stage under control and 2.5 kJ·m^−2^, 5.0 kJ·m^−2^, and 7.5 kJ·m^−2^ UV-B radiation intensity. Bars, 2 cm (white line at the bottom right of leaf angle morphology diagram).

In order to know whether the variation of flag leaf angle is affected by UV-B radiation, we measured the flag leaf angle of rice under different intensities of UV-B radiation. As radiation increased, the angle of flag leaves at the heading stage of rice increased by varying degrees (Figure 1). After the radiation intensity increased to 5.0 kJ·m^−2^, the included angle of the flag leaves began to increase significantly, being 25.6% higher than that of natural light (hereafter as ‘control’). Flag leaves displayed the most significant included angle at radiation intensities of 7.5 kJ·m^−2^, with an increase of 50.2%. Low-intensity UV-B radiation did not significantly affect the included angle of rice flag leaves. However, high-intensity UV-B radiation significantly increased the angle of rice flag leaves.

### 2.2. High-Intensity UV-B Radiation Promotes Cell Elongation and Inhibits Cellulose Synthesis

**Figure 2:**
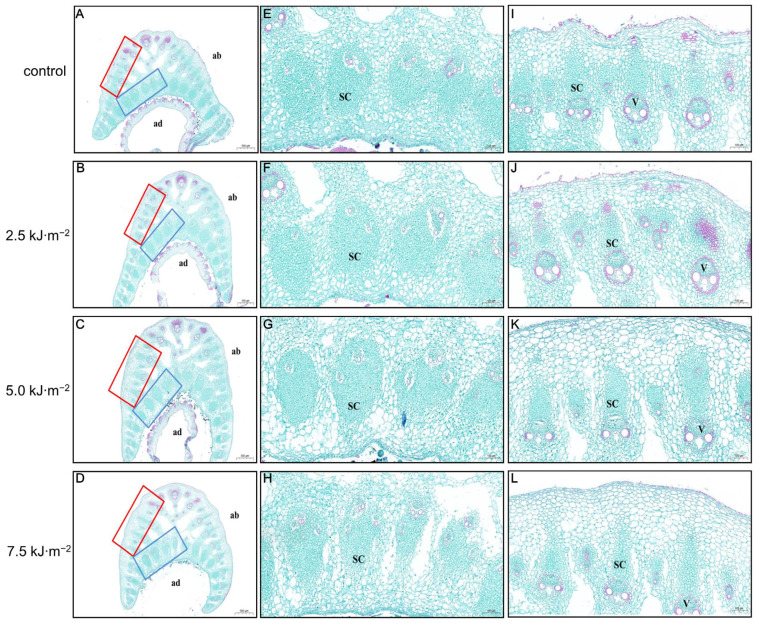
Effect of UV-B radiation on the angle of flag leaves in rice. (**A**–**D**) Complete cross-sections of rice leaf pulvini exposed to natural light (control) and radiation at 2.5 kJ·m^−2^, 5.0 kJ·m^−2^, and 7.5 kJ·m^−2^. ab: abaxial, ad: adaxial. Bars, 500 µm (*n* = 10). The red box represents the abaxial, and the blue box represents the adaxial. (**E**–**H**) Zoomed-in view of the adaxial side of rice leaf pulvini irradiated with natural light (control), 2.5 kJ·m^−2^, 5.0 kJ·m^−2^, and 7.5 kJ·m^−2^ shown in (**A**–**D**). Bars, 100 µm. (**I**–**L**) Zoomed-in view of the abaxial interface of rice leaf pulvini irradiated with natural light (control), 2.5 kJ·m^−2^, 5.0 kJ·m^−2^, and 7.5 kJ·m^−2^ shown in (**A**–**D**). Bars, 100 µm.

Rice leaves extend in accordance with the mechanical strength of the leaf pulvini. According to the cross-sections of rice lamina slices under different UV-B radiation intensities, in contrast with the control, as radiation intensity increased, the rice lamina was loosely arranged, fissures between cells increased, and changes became evident in vascular bundles and sclerenchyma cells (Figure 2A–D). As the radiation intensity increased, the area of lamellar sclerenchyma cells gradually decreased in the enlarged paraxial region of the rice lamina (Figure 2E–H). The 7.5 kJ·m^−2^ UV-B radiation intensity treatments resulted in a significant reduction in the level. As a result of the enlargement of the abaxial axis of the rice lamina (Figure 2I–L), it can be seen that the area of the thick wall cells and vascular bundles of the pulvini under 7.5 kJ·m^−2^ radiation treatment was significantly smaller than that under other treatments.

**Figure 3:**
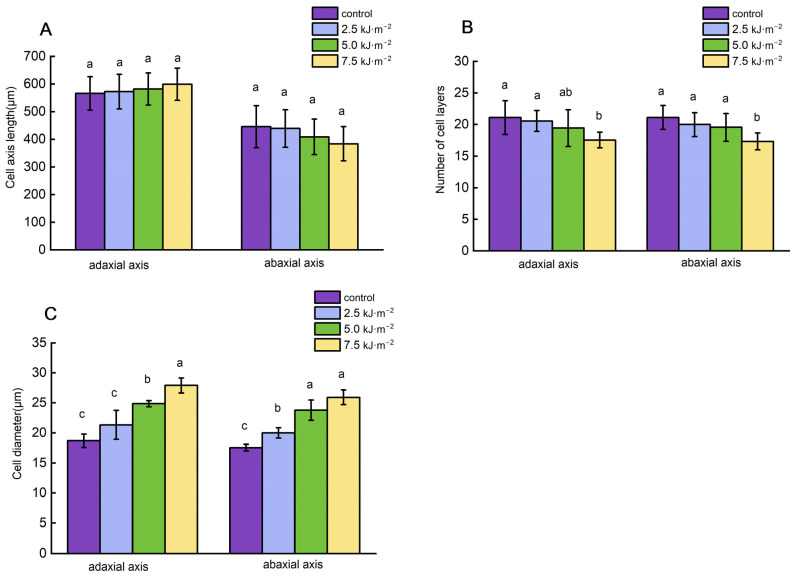
Effects of UV-B radiation on axis length of cells of leaf pulvini in rice. (**A**–**C**) Axial length, cell layer, and cell diameter of leaf pulvini cells in rice treated with natural light (control) and different UV-B radiation intensities (2.5 kJ·m^−2^, 5.0 kJ·m^−2^, and 7.5 kJ·m^−2^) and cell diameter. Error bars are SE (*n* = 10). Different letters indicate significant differences, *p* < 0.05, by one-way ANOVA.

**Figure 4:**
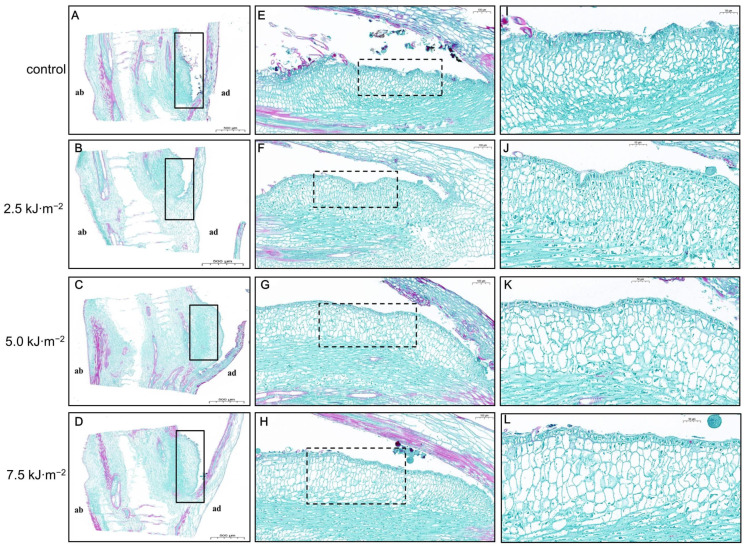
Effects of UV-B radiation on longitudinal structure of leaf pulvini in rice. (**A**–**D**) Longitudinal sections of rice leaf pulvini exposed to natural light (control) and UV-B radiation at 2.5 kJ·m^−2^, 5.0 kJ·m^−2^, and 7.5 kJ·m^−2^ radiation. ab: abaxial, ad: adaxial. Bars, 500 µm (*n* = 10). The black box represents the adaxial section. (**E**–**H**) The dashed black box represents the magnified adaxial section. The 5× magnification of the adaxial section of the rice’s leaf pulvini irradiated with natural light (control) and UV-B radiation at 2.5 kJ·m^−2^, 5.0 kJ·m^−2^, and 7.5 kJ·m^−2^ shown in (**A**–**D**). Bars, 100 µm. (**I**–**L**) The 2× magnification of the adaxial section of the rice’s leaf pulvini shown in (**A**–**D**). Bars, 50 µm.

**Figure 5:**
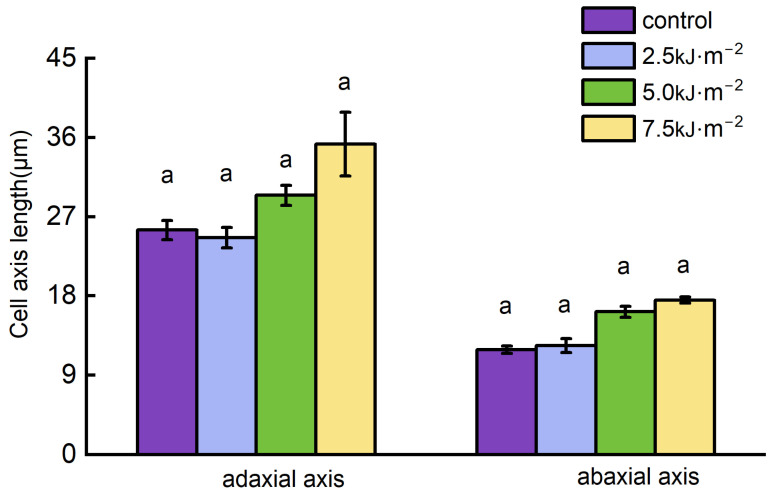
Effects of UV-B radiation on the size of adaxial cells in the longitudinal section of rice leaf pulvini. Cell length and cell width of adaxial cells in rice treated with natural light (control) and different UV-B radiation intensities (2.5 kJ·m^−2^, 5.0 kJ·m^−2^, and 7.5 kJ·m^−2^). Error bars are SE (*n* = 10). Different letters indicate significant differences, *p* < 0.05, by one-way ANOVA.

UV-B radiation had an effect on the number and size of the pulvini cell layers. The changes in the pulvini cell layers decreased as the UV-B radiation intensity increased (Figure 3). When the radiation intensity reached 7.5 kJ·m^−2^, the number of cell layers decreased by 16.8% and 17.9%, respectively, in comparison with natural light (Figure 3B). Additionally, different levels of UV-B radiation resulted in significant changes in the size of lamina parenchyma cells. Adaxial and abaxial parenchyma cells in the pulvini gradually enlarged with increasing radiation intensity. As compared with natural light treatment, 7.5 kJ·m^−2^ irradiation increased the diameters of adaxial and abaxial cells by 49.1% and 47.6%, respectively (Figure 3C). In longitudinal sections of the lamina, the longitudinal cytoarchitectural analysis revealed that UV-B radiation caused paraxial cells to elongate at a certain intensity (Figure 4 and Figure 5). Compared with natural light, the length and width of cells increased to their maximum when the radiation intensity reached 7.5 kJ·m^−2^.

As a result of the increased UV-B radiation, flag leaf pulvini cells are inhibited from dividing and compensate for this loss of cell division by promoting cell expansion. In the longitudinal section of the pulvini, abnormal changes in the size of the abaxial cells suggest that high-intensity UV-B radiation may control the flag leaf angle through the regulation of the longitudinal elongation of the paraxial cells.

**Figure 6:**
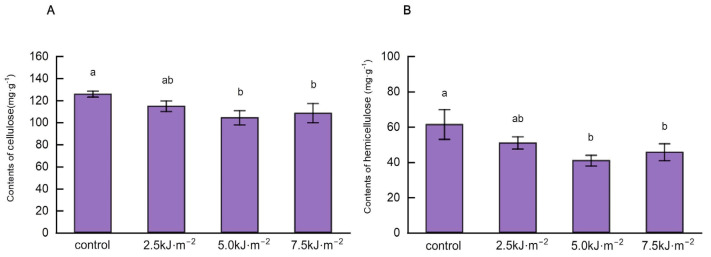
Effect of UV-B radiation on cellulose and hemicellulose content in rice leaf pulvini. (**A**,**B**) The content of cellulose and hemicellulose in rice leaf pulvini treated with natural light (control) and different UV-B radiation intensities (2.5 kJ·m^−2^, 5.0 kJ·m^−2^, and 7.5 kJ·m^−2^). Error bars are SE (*n* = 10). Different letters indicate significant differences, *p* < 0.05, by one-way ANOVA.

We determined the cellulose and hemicellulose content of the leaf pulvini of rice flag leaves treated with additional UV-B radiation using changes in the area of sclerenchyma and the microstructural characteristics of vascular bundles under different UV-B radiation intensities (Figure 6). As radiation levels increased, the amount of cellulose in the lamina decreased. Compared with the natural light treatment, the cellulose content in the leaf pulvini decreased by 13.8% and the hemicellulose content decreased by 33.4%, when the radiation intensity was 5.0 kJ·m^−2^. This result is similar to the anatomical structure of the rice lamina, which means UV-B radiation may reduce the mechanical force of the lamina and adjust the leaf angle by altering the formation of sclerenchyma and the synthesis of cell wall components.

### 2.3. Radiation with High-Intensity UV-B Positively Regulates the BR and GA Content of the Cusp and Negatively Regulates the IAA Content of the Point

**Figure 7:**
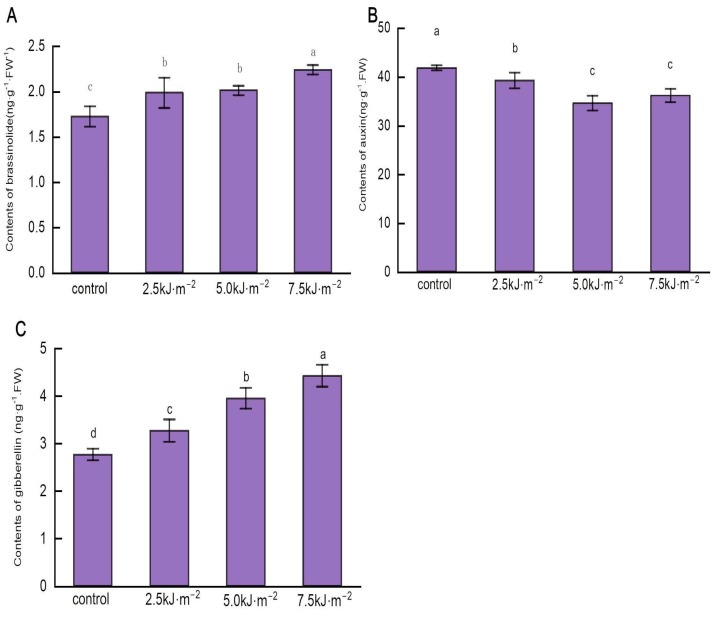
Effect of UV-B radiation on the content of phytohormone in rice leaf pulvini. (**A**–**C**) The content of brassinolide, auxin, and gibberellins in rice leaf pulvini treated with natural light (control) and different UV-B radiation intensities (2.5 kJ·m^−2^, 5.0 kJ·m^−2^, and 7.5 kJ·m^−2^). Error bars are SE (*n* = 10). Different letters indicate significant differences, *p* < 0.05, by one-way ANOVA.

The BR is responsible for controlling the lamina’s development [19]. GA and IAA also contribute to the development of the lamina but usually interact with the BR to determine the leaf angle [20]. High-performance liquid chromatography was used to determine the phytohormone content of the rice leaf pulvini. UV-B radiation increased BR and GA’s range in the leaf pulvini of rice flag leaves. When the radiation intensity reached 7.5 kJ·m^−2^, the BR and GA contents (Figure 7A,C) of the leaf pulvini reached their maximum and rose by 29.94% and 60.1%, respectively, compared with natural light. As a result of increased UV-B radiation, rice lamina auxin content is reduced (Figure 7B). After expanding the radiation intensity to 5.0 kJ·m^−2^, the auxin content in the lamina decreased to the lowest value, 17.3% lower than that of natural light.

### 2.4. UV-B Radiation Changes the Content of Hormone Synthase and Cellulose Synthase

**Figure 8:**
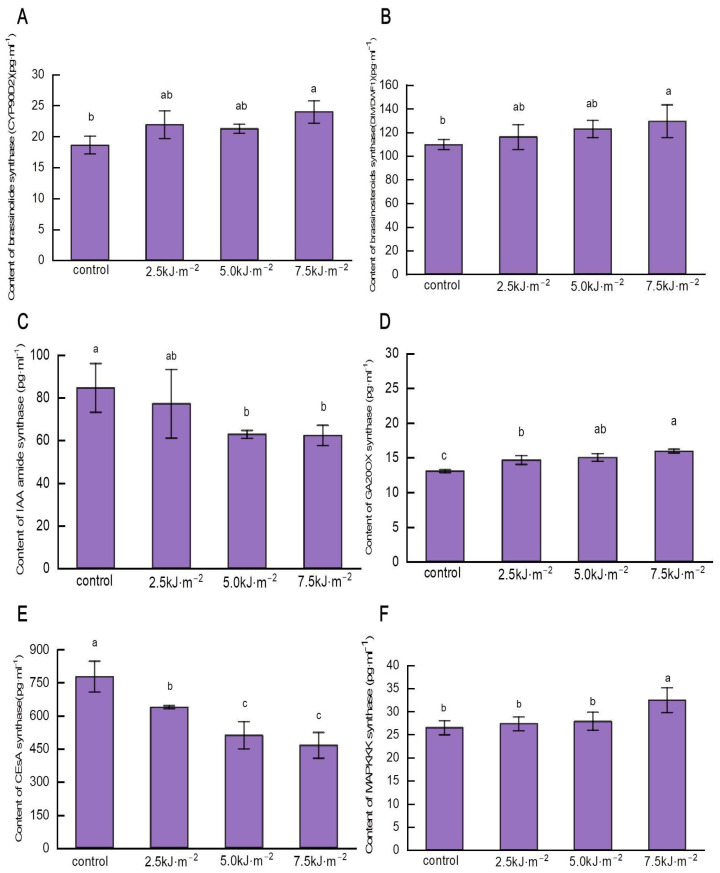
Effects of UV-B radiation on synthase content of leaf pulvini in rice. (**A**) BR biosynthesis enzyme DIM/DWF1 content. (**B**) Brassinolide biosynthesis CYP90D2 content. (**C**) Gibberellin biosynthesis enzyme GA20ox content. (**D**) The content of IAA amide synthase. (**E**) CESA content of cellulose synthase. (**F**) The content of mitogen-activated protein kinase (MAPKKK) encoded by *ILA1*. Error bars are SE (*n* = 10). Different letters indicate significant differences, *p* < 0.05, by one-way ANOVA.

The leaf angle is regulated by plant hormones and cellulose, whose biosynthesis enzymes determine the amount of synthesis. As a result of high-intensity UV-B radiation, the brassinosteroid synthase enzymes CYP90D2, DIM/DWF1, and GA20ox were significantly increased and IAA amide synthase was inhibited. Under 7.5 kJ·m^−2^ radiation treatment, the enzyme contents of CYP90D2, DIM/DWF1, and GA20ox significantly increased by 29.0%, 18.0%, and 22.2%, respectively (Figure 8A–C); IAA amide synthase decreased significantly by 26.4% (Figure 8D).

Enhanced UV-B radiation significantly inhibited the content of CESA enzyme and induced the increase in MAPKKK enzyme content. With the continuous increase in radiation intensity, the CESA enzyme content decreased significantly as compared with natural light (Figure 8E). Compared with natural light, the enzyme content of MAPKKK increased by 22.7% when UV-B radiation reached 7.5 kJ·m^−2^ (Figure 8F). UV-B radiation enhanced hormone and cellulose synthase content to some extent, which, in turn, affected hormone and cellulose synthesis.

### 2.5. UV-B Radiation Alters the Expression of Hormone-Regulated Genes and Mechano-Tissue-Regulated Genes

**Figure 9:**
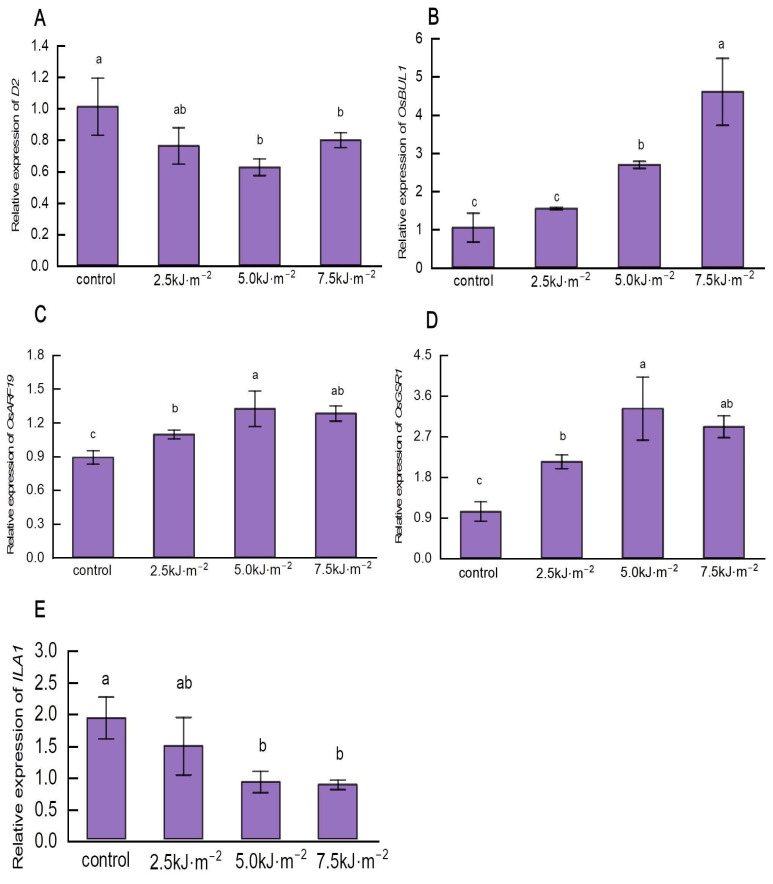
The effect of UV-B radiation on the expression levels of regulated genes in rice lamina. (**A**) The expression level of the BR biosynthesis gene *D2*. (**B**) The expression level of the leaf angle regulatory gene *Oryza sativa BRASSINOSTEROID*. (**C**) The expression level of *UPREGULATED 1-LIKE 1* (*OsBUL1*), which promotes cell elongation in the pulvini. (**D**) The expression level of *OsARF19*, a gene that links IAA and BR signals to regulate the change in the leaf angle. (**E**) The expression level of *OsGSR1*, which interacts with *DIM/DWF1* (*BRD2*) enzymes to activate BR biosynthesis. Error bars are SE (*n* = 10). Different letters indicate significant differences, *p* < 0.05, by one-way ANOVA.

The expression of leaf angle regulating genes under UV-B radiation was analyzed by RT-qPCR, including the hormone biosynthesis genes *D2*, *OsBUL1*, *OsARF19*, and *OsGSR1*, and the gene that controls mechanical tissue formation, *ILA1*. UV-B radiation decreased the expression of BR-regulated gene *D2*. Under ultraviolet-B radiation, *D2* expression decreased to a minimum level that was 38.2% less than natural light (Figure 9A), whereas *OsBUL1* expression increased significantly in response to UV-B radiation (Figure 9B).

*OsARF19* expression increased significantly with the increase in radiation intensity. In contrast to natural light, *OsARF19* expression rose by 48.8% when the radiation intensity was raised to 5.0 kJ·m^−2^ (Figure 9C). Radiation intensity increased first and then decreased the expression of the GA-regulated gene *OsGSR1*. The expression of *OsGSR1* rose to the highest level when the radiation intensity reached 5.0 kJ·m^−2^ and then decreased thereafter (Figure 9D).

The expression of the mechanical tissue regulation gene *ILA1* decreased with increasing radiation intensity. The expression of *ILA1* decreased to its lowest level when the radiation intensity increased to 7.5 kJ·m^−2^, which is 55.0% lower than natural light (Figure 9E). According to the results of RT-qPCR analysis, high-intensity UV-B radiation significantly induced the expression of *OsBUL1*, *OsARF19*, and *OsGSR1*, and significantly inhibited that of *D2* and *ILA1*.

## 3. Discussion

### 3.1. High-Intensity UV-B Radiation May Damage the Rice Tolerance Mechanism When It Promotes Rice Flag Leaf Angle

Even though low-intensity UV-B radiation had no significant effects on the included angles of rice flag leaves, high-intensity UV-B radiation significantly increased the outward expansion of flag leaves, leading to an increase in the included angles. The consensus is that UV-B radiation induces relatively compact structures in plants, but there are several reports on diverse plant phenotypes, which may be the result of differences between species or mechanisms of induction by high- and low-intensity UV-B radiation [35]. The UV-B radiation induces shading in some plants, reducing the penetration of UV-B radiation into the leaves. By flowering and bolting, *Arabidopsis thaliana* balances the adverse effects of shading caused by UV-B radiation [36]. Observations have shown that white clover grows taller and has a flatter canopy after UV-B irradiation, whereas duckweed tends to grow shorter and have an upright structure after UV-B irradiation [37]. In contrast, our study indicated that high-intensity UV-B radiation significantly expanded the included angle of rice flag leaves, which may be an effect of UV-B radiation on different species.

In addition to regulating photomorphogenesis, UV-B can also damage plants by damaging DNA, triggering the accumulation of reactive oxygen species, and inhibiting hypocotyl elongation and cotyledon expansion [38]. Thus, plants can tolerate a certain amount of UV-B radiation. If the tolerance limit is exceeded, plants may expend a significant amount of energy to resist external stresses, resulting in poor growth and development. Under normal growth conditions, *OsCYP84A* was widely expressed; however, under UV-B radiation, it was repressed, resulting in marked growth retardation and obvious signs of plant damage [39]. As a result of high-intensity UV-B radiation treatment, *OsGLP1* knockout rice mutant plants showed an increased leaf angle under solar radiation (including UV-B). *OsGLP1*-deficient mutant plants exhibit lower levels of expression for genes related to flavonoid metabolism—*OsUVR8*, *OsPIF3* and *OsPDX1.2* (pyridoxal 50-phosphate synthase subunit PDX1.2)—and silk exhibits a higher expression level of mitogen-activated protein kinase—OsMPK13 [21,22]. A mutation in *OsGLP1* causes rice plants to be more sensitive to UV-B radiation and reduces the expression of some protective genes, so high-intensity UV-B radiation may cause DNA damage to rice with *OsGLP1-like* genes.

Plants are tolerant to UV radiation when MAPK cascade-related genes are expressed [40]. A variety of cellular activities are mediated by MAPK cascades, including cell division and differentiation, responses to abiotic and biotic stresses, and programmed cell death [41,42]. The MAPK cascade consists of three levels of serine/threonine-specific protein kinases: MAPK, MAPK kinase (MAPKK), and MAPKKK [43]. High-intensity UV-B radiation induced the increase in MAPKKK enzyme content, which may be the stress response of rice leaf pulvini to high-intensity UV-B radiation stress. In the face of UV-B radiation stress, rice also induces the accumulation of anti-UV substances, such as flavonoids and anthocyanins, to resist the effects of UV-B radiation. Our previous research results showed that the enhancement of UV-B radiation in terms of phenolic synthesis manifested as low-intensity promotion and high-intensity inhibition, and the level of phenolic content directly affected the free radical scavenging rate [17]. Enhanced UV-B radiation can promote the synthesis of procyanidins in rice husks, but too high UV-B radiation intensity can inhibit the synthesis of procyanidins in rice husks [15]. High-intensity UV-B radiation may destroy the tolerance system and adaptive mechanism of rice by destroying the DNA of the UV tolerance gene and inhibiting the synthesis of UV-resistant substances, resulting in an increase in the angle between the flag leaves of rice.

### 3.2. Rice Flag Leaf Angle Is Modulated by UV-B Radiation by Affecting Leaf Pulvini Cell’s Development

Cells play a critical role in regulating organ size, and changes in cells directly affect the development of plant organs [44]. The anatomical structure of the leaf pulvini in this study showed that high-intensity UV-B radiation resulted in a decrease in the number of cell layers and an increase in cell size in the cross-section of the pulvini, as well as the significant elongation of the paraxial cells in longitudinal sections. In pulvinus’ adaxial parenchyma cells, expansion and elongation increase the leaf angle [24,25]. High-intensity UV-B radiation may alter the lamellar structure by causing paraxial cells to elongate longitudinally, increasing the inclusion angle of rice flag leaves. To initiate cellular responses to external stimuli, UV-B radiation-induced morphogenesis requires complex hormonal signaling mechanisms [45]. A decrease in gibberellin levels caused by UV-B radiation suppresses leaf growth, allowing plants to take advantage of shortened growth zones and decreased cell yield [46]. The effect of UV-B radiation on the production of IAA in *Arabidopsis* roots is inhibited, the accumulation of IAA is reduced, and the unbalanced development of *Arabidopsis* root cells results in root bending [47]. Based on a correlation analysis of plant hormones with flag leaf angle under UV-B radiation, the following conclusions were drawn: brassinolide and gibberellin content in rice leaf pulvini was significantly positively correlated with flag leaf angle, and rice leaf pulvini growth was significantly correlated with flag leaf angle. The angle of the flag leaf and the element content were significantly negatively correlated. UV-B radiation regulates the angle of rice flag leaves by controlling hormones, which is consistent with the change law of hormone content and leaf angle.

Rice genes that are regulated by UV-B radiation are also related to phytohormones [48]. After UV-B radiation treatment, the *OsBUL1* gene was highly expressed in the leaf pulvini of rice flag leaves. In response to UV-B radiation, *OsBUL1* may be a key gene responsible for determining the angle of rice flag leaves. High-intensity UV-B radiation inhibited the expression of *D2* but increased the concentration of CYP90D2. UV-B radiation may not regulate the CYP90D2 enzyme through the *D2* gene, but will stimulate it to synthesize BR through other potential genes, and will regulate the structure of flag leaf pulvini cells so that the angle of the flag leaf is enhanced. In addition to upregulating the expression of *OsGSR1* and *OsARF19*, UV-B radiation also increased the level of GA20ox enzymes and decreased the content of IAA amide synthase. UV-B radiation regulates flag leaf angle through *OsGSR1* and *OsARF19*.

The *OsGSR1* and *OsARF19* genes are also involved in the synthesis of BR. As part of the BR biosynthesis pathway, *OsGSR1* interacts with the enzyme DIM/DWF1 (BRD2), an enzyme responsible for catalyzing the conversion of 24-methylene cholesterol to campesterol [33]. High-intensity UV-B radiation can increase DIM/DWF1 enzyme content, and the OsGSR1 gene is involved in the synthesis of BR. The BR biosynthesis genes *CYP90D2* and *OsDWARF* are upregulated in plants overexpressing *OsARF19-O1* [19,23,32]. Therefore, high-intensity UV-B radiation may mediate BR synthesis by DIM/DWF1 and CYP90D2 enzymes through the *OsGSR1* and *OsARF19* genes.

### 3.3. Rice Flag Leaves Are Modulated by UV-B Radiation through a Mechanical Effect on the Leaf Pulvini

In addition to creating lateral junctions between the structural polymers of needle leaf structures of spruce (*Picea omorika*), UV-B radiation also changes the mechanical properties of the needle leaf wall, resulting in reductions in lignin and cellulose contents, as well as changes in cellulose crystallinity [49]. High-intensity UV-B radiation inhibits cellulose and hemicellulose contents in rice leaf pulvini. In the pulvini, there was a reduction in the amount of sclerenchyma and vascular bundles, as well as a decrease in mechanical strength. According to these results, UV-B radiation stress results in changes in the mechanical properties of rice leaf pulvini.

*ILA1* regulates pulvini mechanical organization by interacting with a family of nucleoproteins (*IIPs*). UV-B radiation reduced the content of the CESA enzyme and inhibited the expression of the *ILA1* gene. Mutants lacking *ILA1* have smaller vascular bundles and reduced cellulose, which weakens the mechanical support of the cusp [34]. In plants overexpressing *OsARF19*, it was also found that the content of cellulose, the main component of the lamellar sclerenchyma cell wall, was decreased, and the leaf angle was significantly increased [31]. The correlation analysis of cellulose and hemicellulose contents in rice flag leaf pulvini and flag leaf angle showed that cellulose and hemicellulose contents in rice flag leaf pulvini under UV-B radiation were significantly negatively correlated with flag leaf angle. It can be speculated that high-intensity UV-B radiation will induce the expression of the mechano-regulated rice gene *ILA1*, weaken the mechanical support of the lamina to the leaf by changing the cell wall components and mechanical organization of the lamina, and then lead to the expansion of the rice flag leaf in the abaxial direction.

### 3.4. Linkage between the Phytohormonal Pathway and the Mechanical Support Pathway in Regulating the Flag Leaf Angle of Rice under UV-B Radiation

**Figure 10:**
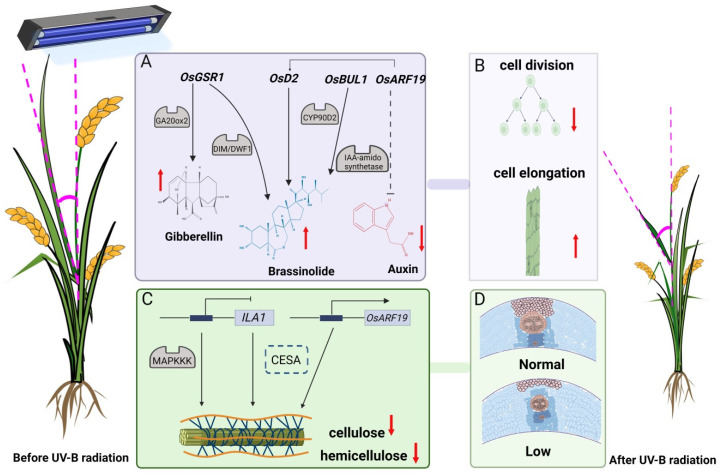
Model of UV-B radiation regulation of rice flag leaf angle. ↓ indicates positive regulation and ⊥ indicates negative regulation. Solid arrows indicate direct regulation and dashed arrows indicate indirect regulation. (**A**) *OsBUL1*, *OsGSR1*, and *OsARF19* regulate leaf pinch angle through the phytohormone pathway. (**B**) Elongation of leaf pulvini cells and inhibition of cell division. (**C**) *ILA1* and *OsARF19* regulate leaf pinch angle through the mechanical support pathway. Dashed blue boxes indicate interactions that changed and were not experimentally verified. (**D**) The strength of leaf pulvini mechanical support is reduced.

Under high UV-B radiation intensity, *OsBUL1*, *OsGSR1*, and *OsARF19* regulate flag leaf angle, regulate and promote the elongation of pulvinus’ cells, inhibit cell division, and positively regulate flag leaf angle through plant hormone pathways (BR, GA, and IAA). In the mechanical support pathway, UV-B inhibits the expression of *ILA1*, which encodes MAPKKK, interacts with *IIPs* to regulate the mechanical tissue of the pulvinus, decreases the cellulose level; *OsARF19* was overexpressed and the cellulose content decreased. Figure 10 presents a possible model of the overall process, demonstrating that the auxin response gene *OsARF19* deserves attention.

The OsARF19 as an upstream regulating factor of *OsGH3-5* up-regulates *OsGH3-5* expression. The content of free IAA in the leaf pulvini of *OsARF19-O1* and *OsARF19-O2* overexpression lines decreases, which regulates the growth of adaxial/abaxial cells in plant leaves and controls the leaf angle of rice. *OsARF19* directly binds the *OsBRI1* promoter and controls the expression of *OsBRI1* through three different primers, participating in BR signal regulation. In the *OsARF19-O1* and *OsARF19-O2* strains, cell division in the pulvini was enhanced, and compared with *WT/NIP* plants, the area of the vascular bundle was reduced, and the cellulose content was reduced. This result is similar to that of the *ILA1* regulation [31]. The mechanical strength of the pulvini of the double mutant *osarf6osarf17* was significantly reduced, the secondary cell wall components of sclerenchyma cells were reduced, and the flag leaf angle was increased. *OsARF6* and *OsARF17* regulate the expression of the downstream gene *ILA1*, regulate the synthesis of the secondary cell wall of the sclerenchyma cells at the flag leaf node, and then regulate the flag leaf angle [50]. This suggests that, under UV-B radiation, plant hormone signaling and morphological mechanical support pathways are coordinated in leaf angle regulation, and the flag leaf angle of rice is subject to multi-level regulation.

## 4. Materials and Methods

### 4.1. Growing Conditions and Plant Material

The rice variety used in this study was white-footed old japonica, traditionally grown in Yuanyang terraced fields in Honghe Prefecture, Yunnan Province. Planting was conducted at Qingkou Village, Xinjie Town, Yuanyang County, Honghe Prefecture, Yunnan Province, in summer (23°07′15.8′′ N, 102°44′45.6′′ E); the altitude of the field is 1600 m, and the background radiation intensity is ten kJ·m^−2^·d^−^^1^.

### 4.2. UV-B Radiation Treatment

The rice irradiation treatment was set up with four groups of natural light and UV-B radiation (2.5, 5.0, and 7.5 kJ·m^−2^), corresponding to 0%, 10%, 20%, and 30% local ozone attenuation, respectively. A height-adjustable lamp holder was installed above each row of rice, UV-B lamps (40 W, wavelength 280–320 nm) were erected, and the UV-C band light below 280 nm was filtered with 0.13 mm cellulose acetate membrane. The UV-B lamp was placed approximately 60 cm, 40 cm, and 20 cm away from the top of the plant, and the radiation intensity at the top of the rice plant was measured with a UV-B radiometer, and the height of the lampstand was adjusted with the growth of the rice to keep the radiation intensity constant. The lamp irradiation was carried out after rice transplantation and greening to the mature harvest period; the daily irradiation time was set at 7 h (10:00–17:00); and UV-B radiation treatment was not carried out on cloudy or rainy days.

### 4.3. Measurement of the Flag Leaf Angle

Photographs of rice flag leaf blade angles were taken in the field one week after rice tasseling. Leaf angles were measured with the aid of the ImageJ program (*National Institutes of Health. U.S. Department of Health and Human Services*. Available at: https://imagej.nih.gov/ij/ accessed on 23 October 2022), which measures leaf angles in terms of the angle between the rice spike internode and the leaf blade, for at least 20 individual plants.

### 4.4. Preparation of Rice Leaf Pulvini Paraffin Sections

Ten rice flag leaf pulvini were collected from each treatment at the heading stage, fixed with 70% FAA fixative, dehydrated with ethanol, and then transparentized with anhydrous ethanol and xylene. We dipped the tissue in wax, stereotyped it, and sliced it with a microtome at a thickness of 10 mm. A mixture of xylene and absolute ethanol in different proportions was used to dewax the sections, rehydrated with ethanol, and stained with 1% safranin. The concentrations of ethanol solutions were dehydrated, stained with fast green (0.5%), then dehydrated in anhydrous ethanol, and cleared with xylene. The mounting medium should be added dropwise and dried. Lastly, the slices were scanned using digital imaging software (Pannoramic MIDI, Pannoramic 250FLASH, Pannoramic DESK; manufacturer: 3DHISTECH (Budapest, Hungary)).

### 4.5. Determination of Cellulose and Phytohormone Contents, and Their Synthase Activity in Leaf Pulvini

Ten fresh flag leaf pulvini were collected from each treatment at the heading stage of rice and quickly stored in liquid nitrogen for testing. Anthrone analysis was used to determine the cellulose content [34]. Analysis of the phytohormone content of frozen flag leaf pulvini was carried out as follows: The flag leaf pulvini were ground into a fine powder. Methanol was used to extract the powder, and the powder was then ultrasonically treated for 30 min. Following extraction, the material was dried at 50 °C for a week and then dissolved in 5 mL of methanol and centrifuged at 8000 rpm for 25 min. Afterward, the supernatant was filtered through a 0.22 µM membrane filter and analyzed by HPLC (Waters, C18 silica column, reverse phase, Australia). Flow rates of 0.8 mL/min-1 and injection volumes of 20 L were used in HPLC analysis experiments conducted in methanol:water (50:50) [21]. The powder of flag leaf pulvini was mixed with PBS buffer (0.1 mol/L, pH 7.4).The supernatant was collected by centrifugation for analysis using the plant brassinosteroid synthetase DWARF1 (DIM/DWF1) enzyme-linked immunosorbent assay(ELISA) kit, plant brassinolide synthase (CYP90D2) enzyme-linked immunosorbent assay (ELISA) kit, plant IAA amide synthetase (IAAS) enzyme-linked immunosorbent assay (ELISA) kit, plant gibberellin 20 oxidase (GA20OX) enzyme-linked immunosorbent assay (ELISA) kit, plant mitogen-activated protein kinase kinase kinase (MAPKKK) enzyme-linked immunosorbent assay (ELISA) kit, and plant cellulose synthase (CEsA) enzyme-linked immunosorbent assay (ELISA) kit (Grace Biotechnology Co., Ltd., Suzhou, China) [51].

$$\mathrm{Sample}\;\mathrm{Concentration}=\frac{\mathrm{Sample}\;\mathrm{Area}}{\mathrm{Mean}\;\mathrm{Standard}\;\mathrm{Area}}\times \frac{\mathrm{Standard}\;\mathrm{Weight}}{\mathrm{Standard}\;\mathrm{Dilution}}\times \frac{\mathrm{Sample}\;\mathrm{Dilution}}{\mathrm{Sample}\;\mathrm{Weight}}$$

### 4.6. RNA Extraction and RT-qPCR

The tissue samples used in this experiment were taken from the flag leaf pulvini at the heading stage of rice. Ten fresh leaf pulvini from 10 rice plants were pooled together to form a biological replicate, and three biological replicates were analyzed for RNA extraction. To extract RNA from tissue samples, the tissue samples were ground into powder in liquid nitrogen; then, Trizol reagent, chloroform, phenol form, isopropanol, and RNA-free water were added. The Novizan reverse transcription kit (R223) was used to synthesize cDNA according to the manufacturer’s instructions. The expression of *ILA1* was detected by RT-qPCR amplification using *TBC1 domain family member 22A* as a reference gene. In this study, we used quantitative fluorescence PCR (ABI step one plus9 from ABI Company in the United States), and Novozyme RT-qPCR reagent (Q341) as the fluorescence quantitative PCR reagent. To achieve RT-qPCR amplification, the following conditions were used: 95 °C—90 s, 40 cycles (95 °C—5 s, 60 °C—15 s, and 72 °C—20 s). The data presented are the mean of at least two biological replicates with SE. The gene primers are shown in Table 1 and can be obtained from the NCBI database (*National Center for Biotechnology Information. U.S. National Library of Medicine*. Available at: http://www.ncbi.nlm.nih.gov/, accessed on 23 October 2022).

### 4.7. Statistical Analysis

The angle of the rice leaves was measured with the ImageJ1.44 software. Statistical analyses were carried out using SPSS version 21.0 (SPSS Inc., Chicago, IL, USA). The significance among the treatments was determined by a one-way analysis of variance (ANOVA) followed by Duncan’s test (*p* < 0.05). The figures were made using Origin 2022b (OriginLab Inc., Northampton, UK).

## Figures and Tables

**Table 1:** Primers used in this study. Sequence data from this article can be found in the GenBank/EMBL or RiceGE databases under the following accession numbers: *ILA1* (AK073747); *OsARF19* (AK103312); *D2*/*CYP90D2* (A P003244); *OsBUL1* (Os02g51320); and *OsGSR1* (AY604180).

Gene	5′--3′	Tm (°C)	Product (bp)
*ILAL-F*	GCGGAAGGTCAGGAGAGAAC	58.6	142
*ILAL-R*	ACTGGCGTTGAAGCGGTG	59.9	
*D2-F*	TGGAGGTGGAAGGAGAAGGA	59.2	172
*D2-R*	TGGGGAAGTTGACGATGTGG	61.1	
*OsBUL1-F*	GGTTTTTCCCTCTCCCTTCCA	62	91
*OsBUL1-R*	GTAGTGGTCGGTCGGTTGTC	57.4	
*OsARF19-F*	CGAAGGATGCCCAGCAAGAA	63.4	348
*OsARF19-R*	CGTCGCCAAGAAGTAGGATGT	59.4	
*OsGSR1-F*	CTGCCCCTGCTACAACAACT	57.7	296
*OsGSR1-R*	CTCACTCTCCTCTCTCGCAC	54.2	
*TBC1*	TGGTCATGTTCCTTCAGCAC		

## Data Availability

The original contributions presented in the study are included in the article. Further inquiries can be directed to the corresponding author.
